# Supplementary material for: Stratocumulus cloud clearings: statistics from satellites, reanalysis models, and airborne measurements
Source: Atmos Chem Phys. Author manuscript; Available in PMC 2020 Nov 12. (PMC7660233; doi:10.5194/acp-20-4637-2020)
Supplement: supplement [file NIHMS1643613-supplement-supplement.docx]

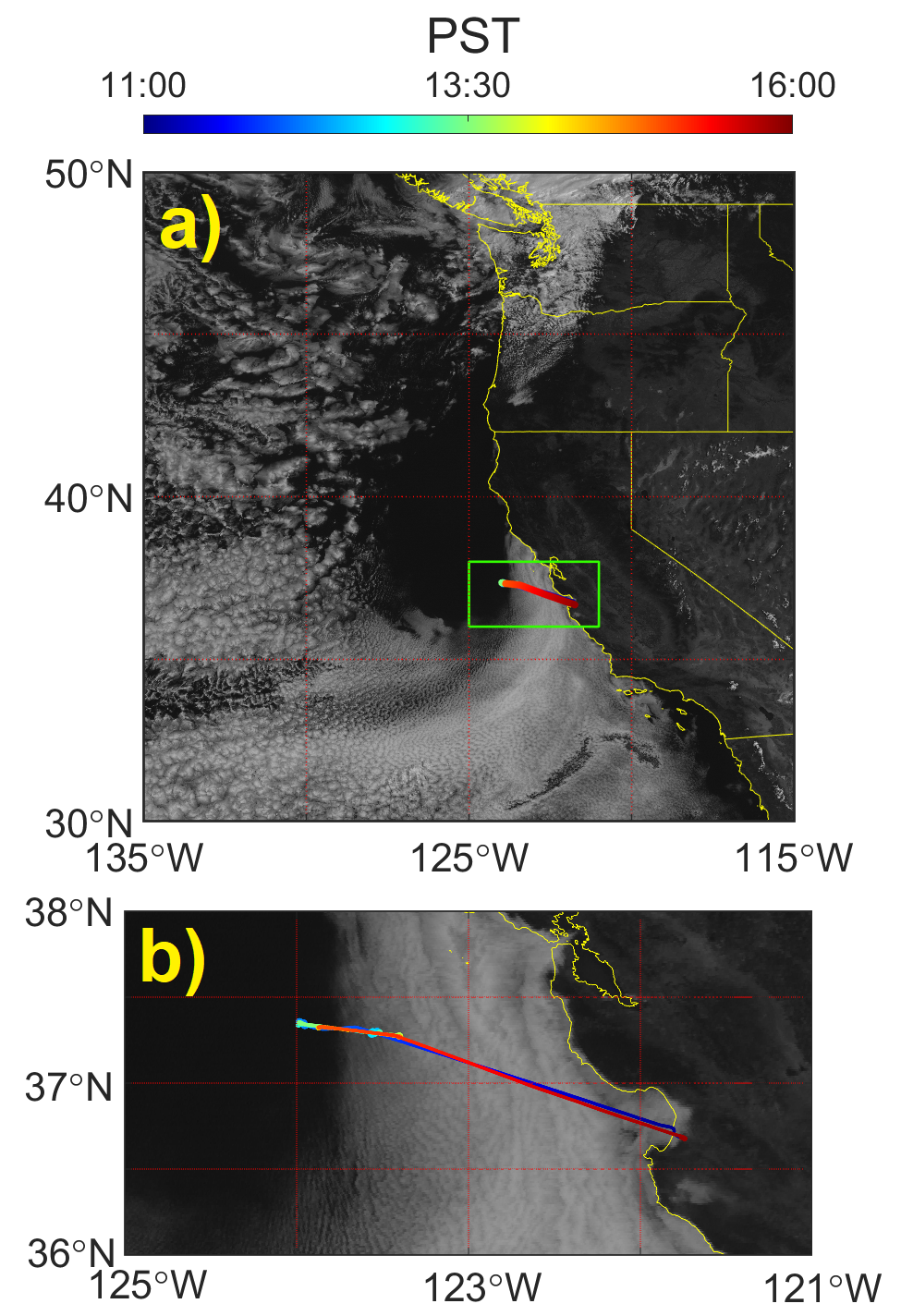


**Figure S1.** a) GOES 15 visible band image (11:45 PST (18:45 UTC) on 02 Aug 2016) with the overlaid flight path of FASE RF08. b) Zoomed in view of the satellite image to highlight the clear-cloudy border.


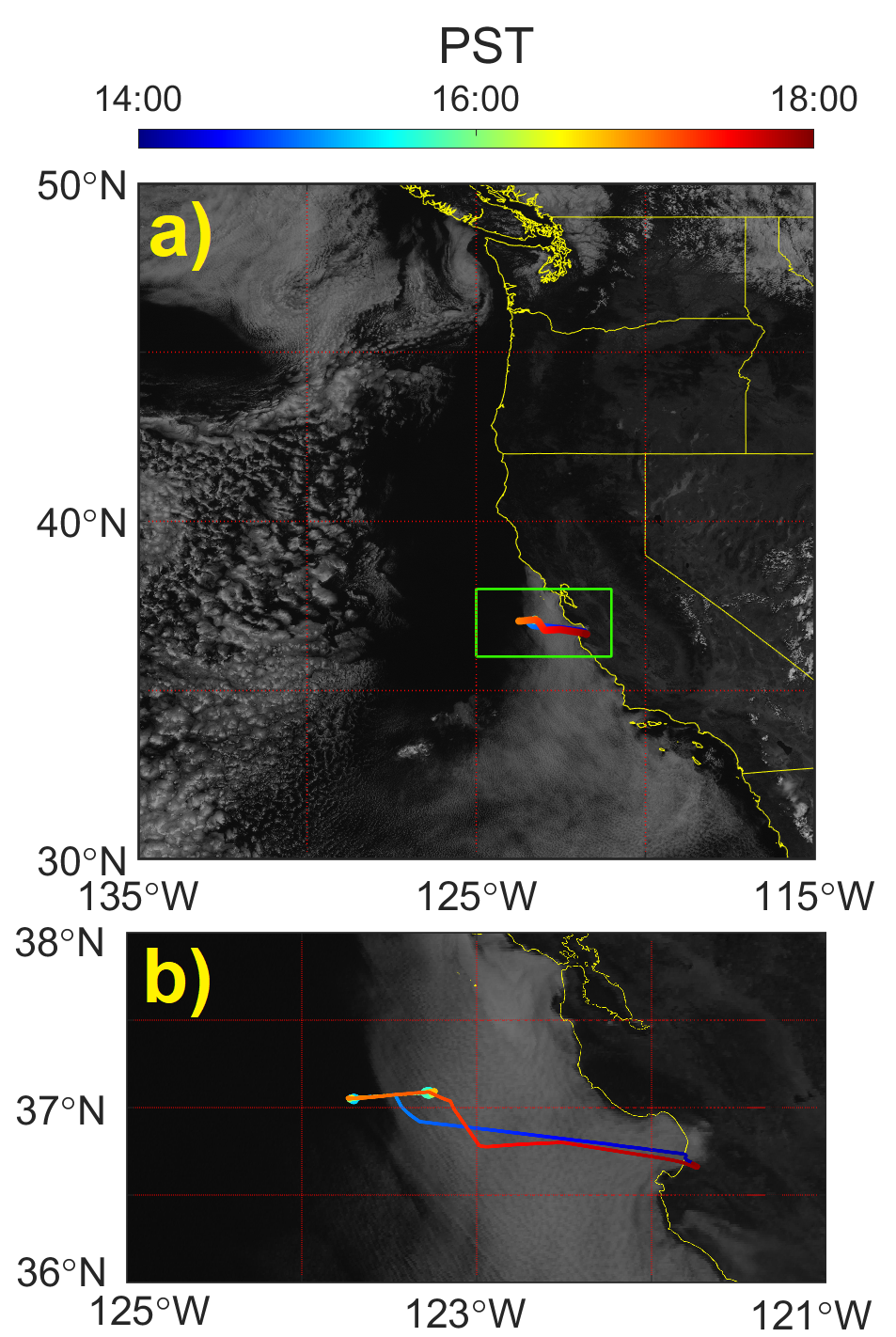


**Figure S2.** Same as Figure S1 except for FASE RF09B (image from 15:15 PST (22:15 UTC) on 03 Aug 2016).


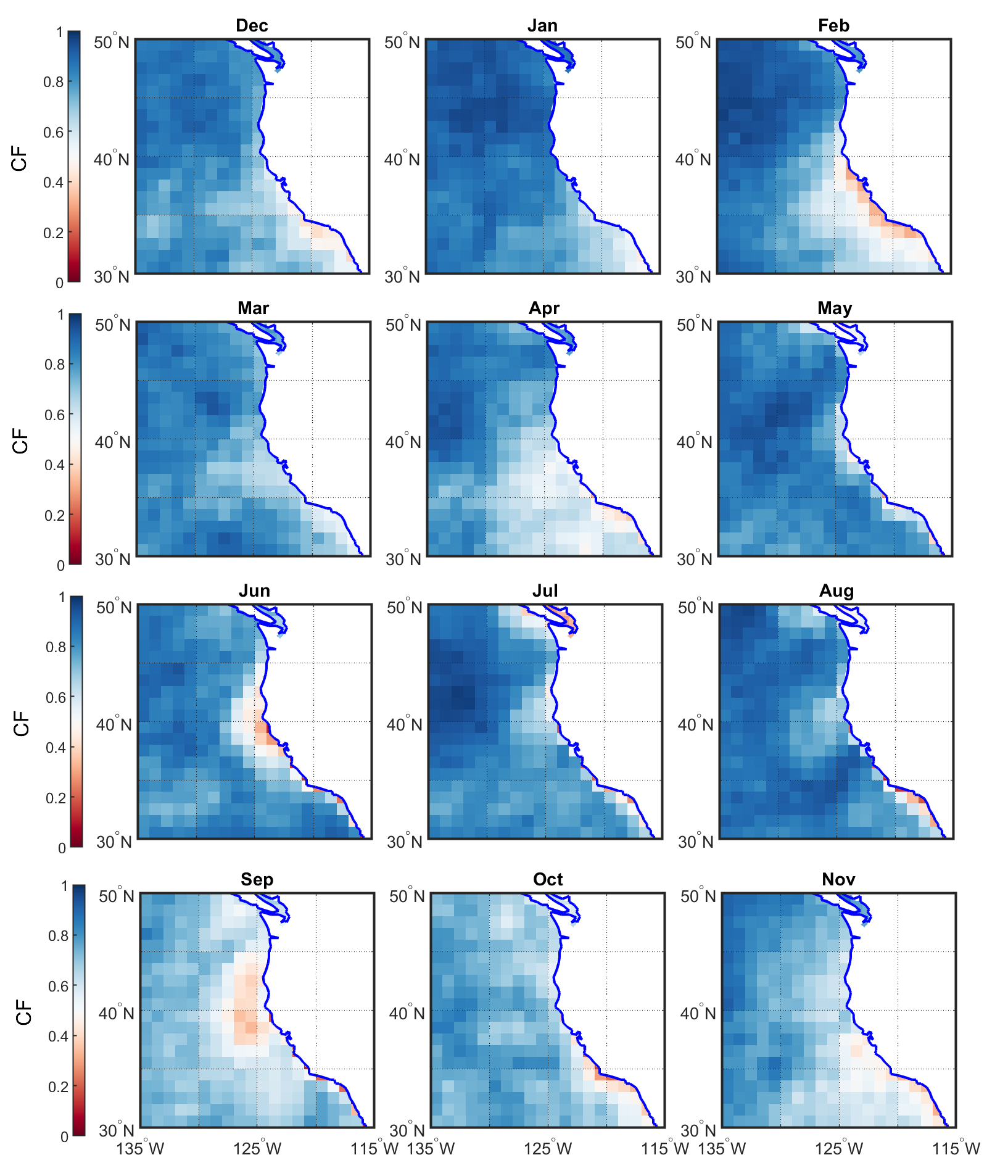


**Figure S3.** Average monthly cloud fraction (*CF*) for 2018 obtained from MODIS Terra Level 3 (Collection 6.1).


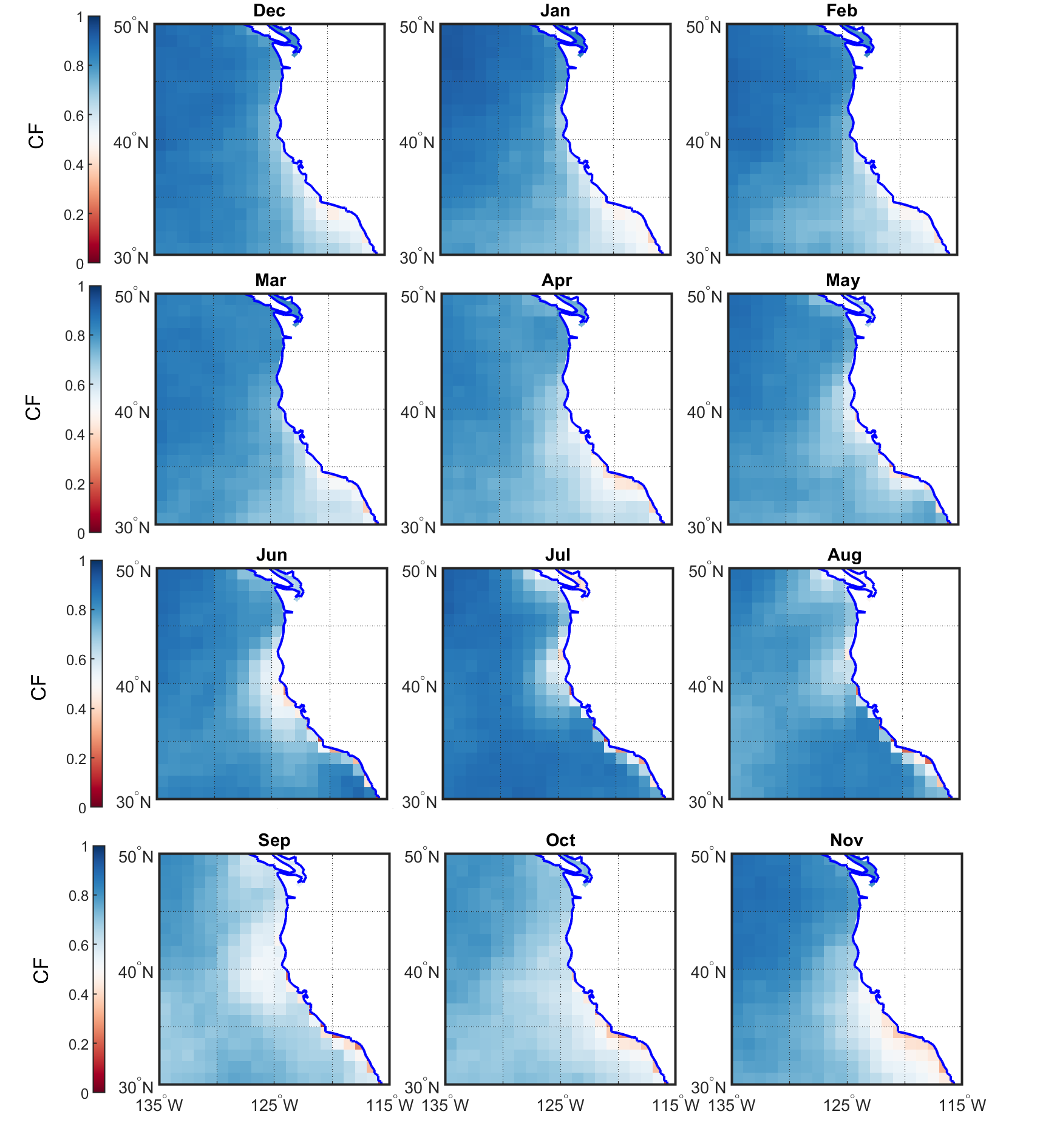


**Figure S4.** Average monthly cloud fraction (*CF*) for 2009 through 2018 obtained from MODIS Terra Level 3 (Collection 6.1).


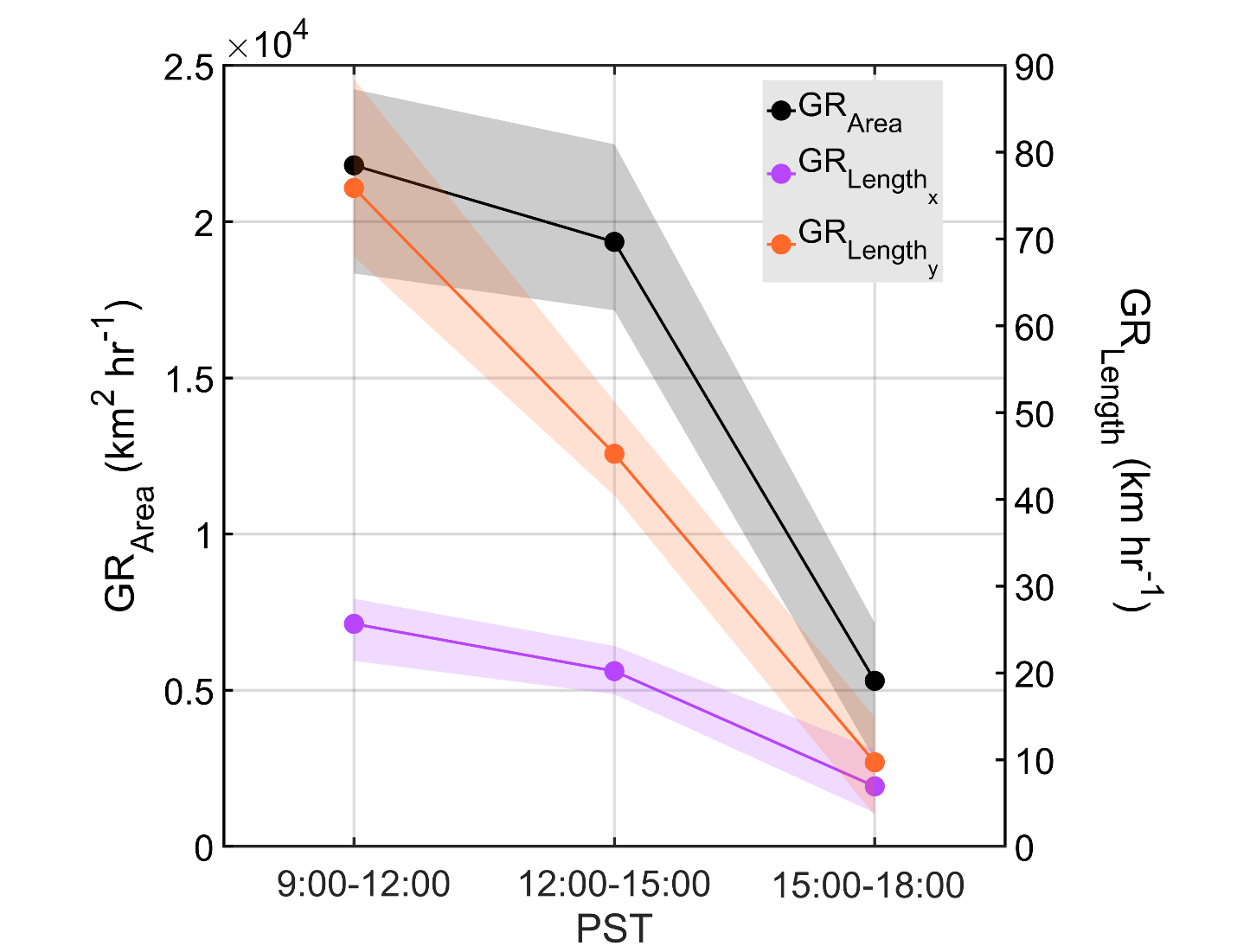


**Figure S5.** The median growth rate of clearing area (left y-axis) and width and length of clearings (right y-axis) over three hour increments. Shadings of curves represent 95% confidence intervals calculated using bootstrapping (n =10,000).


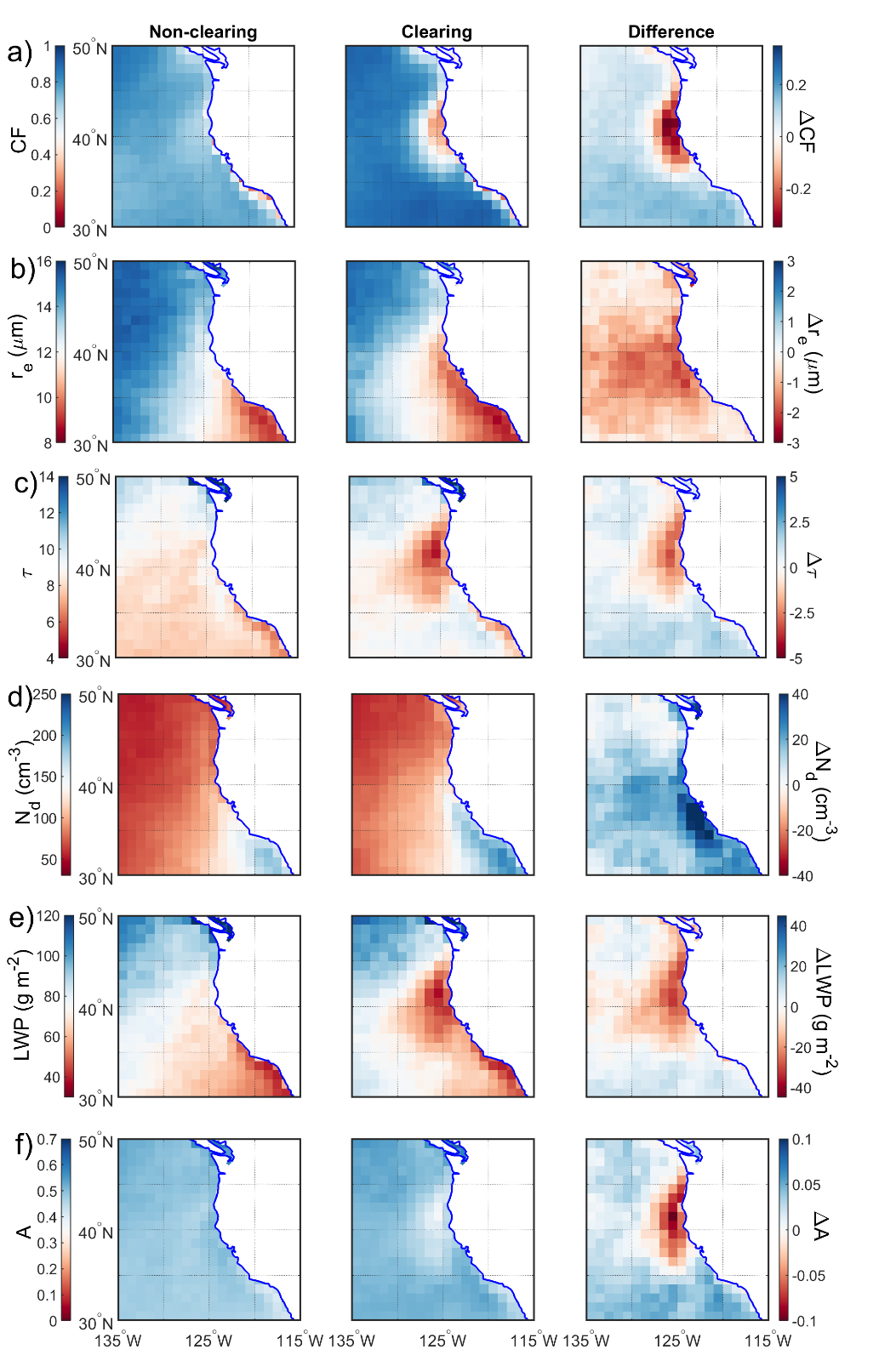


**Figure S6.** Average cloud parameters for non-clearing and clearing days obtained from MODIS Aqua Level 3 (Collection 6.1) data: a) cloud fraction day (*CF*), b) cloud top droplet effective radius (*r_e_*), c) cloud optical thickness (*τ*), d) cloud droplet number concentration (*N_d_*), e) cloud liquid water path (*LWP*), and f) cloud albedo (*A*). Differences (clearing minus non-clearing) are shown in the farthest right column with separate color scales. Values from any instances of clear pixels were omitted from the analysis to produce these figures.


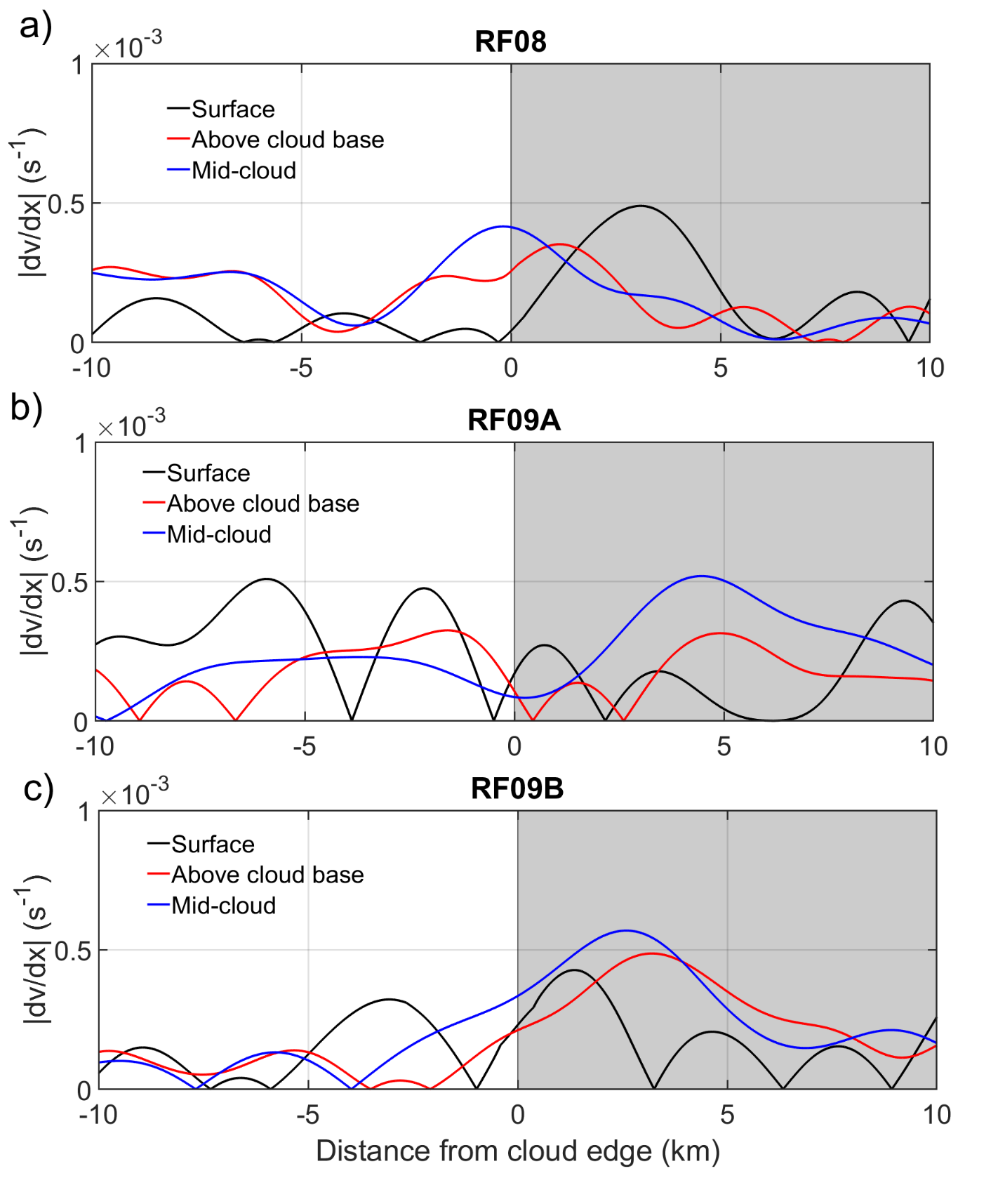


**Figure S7.** Absolute variations in horizontal shear as a function of distance from the cloud boundary for the parallel component of horizontal wind speed for three case research flights: a) RF08, b) RF09A, and c) RF09B. These variations were shown only for constant altitude legs (surface, above cloud base, and mid-cloud legs). Cloudy columns are highlighted in grey.


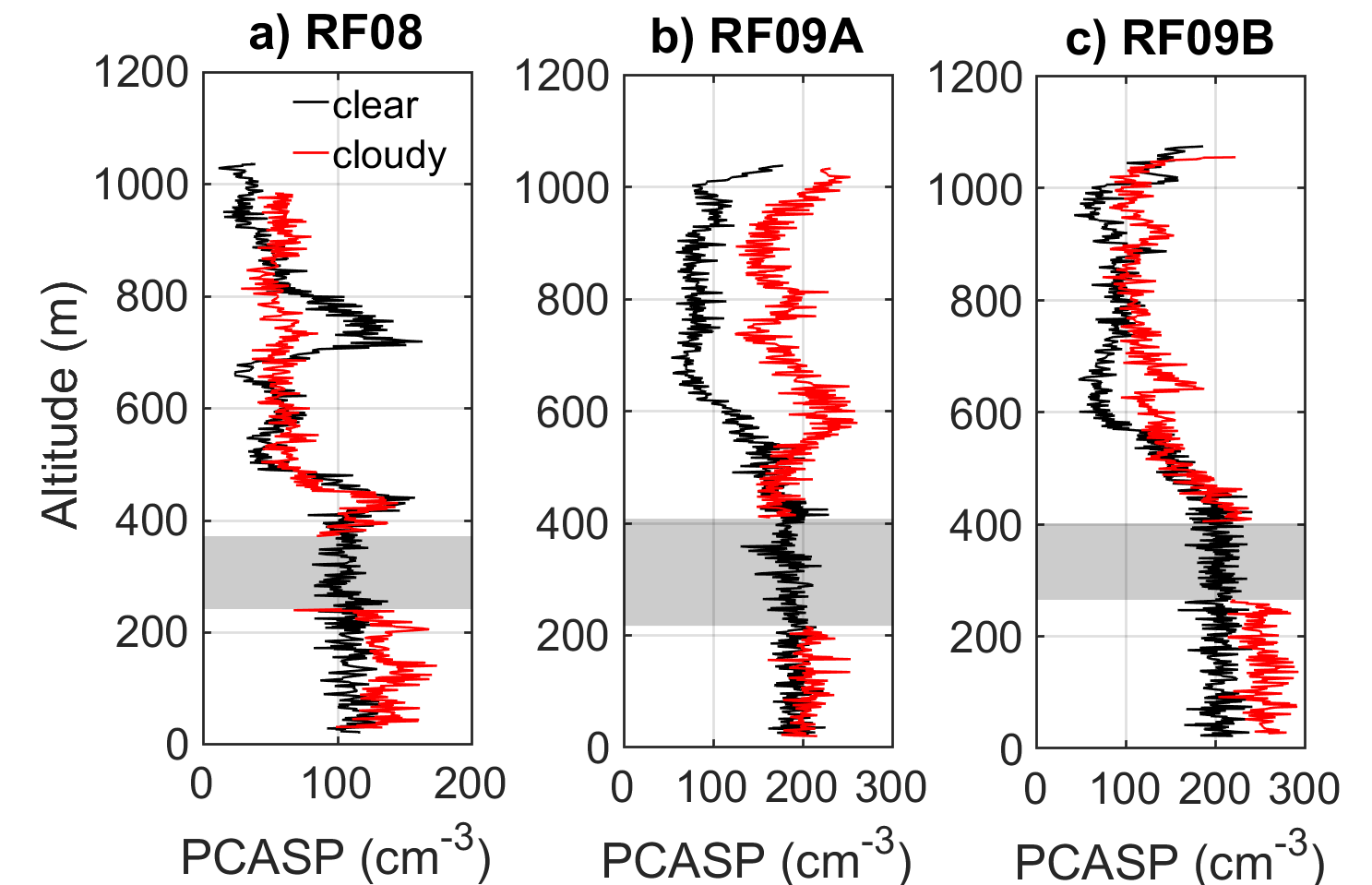


**Figure S8.** PCASP aerosol number concentration profiles obtained from soundings performed in clear and cloudy columns for three case research flights: a) RF08, b) RF09A, and c) RF09B. The altitude range where the cloud deck was present is highlited in grey. PCASP data are unreliable in cloud due to droplet shatter artifacts and thus not shown.
